# Microglial Cells Are Involved in the Susceptibility of NADPH Oxidase Knockout Mice to 6-Hydroxy-Dopamine-Induced Neurodegeneration

**DOI:** 10.1371/journal.pone.0075532

**Published:** 2013-09-23

**Authors:** Marina S. Hernandes, Graziella D. R. Santos, Cecília C. Café-Mendes, Larissa S. Lima, Cristoforo Scavone, Carolina D. Munhoz, Luiz R. G. Britto

**Affiliations:** 1 Department of Physiology and Biophysics, Institute of Biomedical Sciences, University of São Paulo, São Paulo, Brazil; 2 Department of Pharmacology, Institute of Biomedical Sciences, University of São Paulo, São Paulo, Brazil; University of Cologne, Germany

## Abstract

We explored the impact of Nox-2 in modulating inflammatory-mediated microglial responses in the 6-hydroxydopamine (6-OHDA)-induced Parkinson’s disease (PD) model. Nox1 and Nox2 gene expression were found to increase in striatum, whereas a marked increase of Nox2 expression was observed in substantia nigra (SN) of wild-type (wt) mice after PD induction. Gp91^phox-/-^ 6-OHDA-lesioned mice exhibited a significant reduction in the apomorphine-induced rotational behavior, when compared to wt mice. Immunolabeling assays indicated that striatal 6-OHDA injections reduced the number of dopaminergic (DA) neurons in the SN of wt mice. In gp91^phox-/-^ 6-OHDA-lesioned mice the DA degeneration was negligible, suggesting an involvement of Nox in 6-OHDA-mediated SN degeneration. Gp91^phox-/-^ 6-OHDA-lesioned mice treated with minocycline, a tetracycline derivative that exerts multiple anti-inflammatory effects, including microglial inhibition, exhibited increased apomorphine-induced rotational behavior and degeneration of DA neurons after 6-OHDA injections. The same treatment also increased TNF-α release and potentiated NF-κB activation in the SN of gp91^phox-/-^-lesioned mice. Our results demonstrate for the first time that inhibition of microglial cells increases the susceptibility of gp91^phox-/-^ 6-OHDA lesioned mice to develop PD. Blockade of microglia leads to NF-κB activation and TNF-α release into the SN of gp91^phox-/-^ 6-OHDA lesioned mice, a likely mechanism whereby gp91^phox-/-^ 6-OHDA lesioned mice may be more susceptible to develop PD after microglial cell inhibition. Nox2 adds an essential level of regulation to signaling pathways underlying the inflammatory response after PD induction.

## Introduction

NADPH oxidases (Noxes) are multi-subunit enzymes able to transfer electrons across biological membranes, catalyzing the reduction of oxygen to O_2_^·−^ (superoxide) at the expense of NADPH. Superoxide is the primary product of the electron transfer, but other downstream reactive oxygen species (ROS), such as hydrogen peroxide (H_2_O_2_), can also be generated [1,2]. Considering the high susceptibility of the nervous tissue to oxidative damage, the expression of a specialized enzymatic system able to produce ROS in the brain not as a byproduct, but rather as the primary function of the enzymatic complex, was considered unlikely for a long time [3]. However, Nox family members and the ROS they generate have been identified as important contributors to the regulation of physiological and pathological events in the nervous system [3-5]. Seven Nox isoforms have been identified so far: Nox1, gp91^phox^ (Nox2), Nox3, Nox4, Nox5, and Dual Oxidases 1 and 2 (Duox1 and Duox2). Expression of each of them varies according to different tissues and species. In the central nervous system, the presence of Nox1, Nox2, Nox3, and Nox4 isoforms has been identified in several brain structures [6]. The misregulation of Nox isoforms has been linked to a variety of neurodegenerative conditions such as Alzheimer’s and Parkinson’s diseases (PD) [7-10] and, as a consequence, these enzymes have been proposed as a potential pharmacological target for slowing disease progression [11]. However, the mechanisms involved are complex and still incompletely understood. In relation to Parkinson’s disease (PD), a neurodegenerative disorder characterized by the progressive loss of dopaminergic (DA) neurons of the nigrostriatal pathway of the brain, increasing evidence has suggested the involvement of oxidative stress caused by overactivation of this enzymatic system on its pathogenesis [12,13]. Recently, Nox1expression was found to be increased in the substantia nigra (SN) of PD patients, suggesting that the Nox complex plays a role in the degeneration of those neurons. In addition, the genetic intervention on Nox1 and its chemical inhibition protected nuclear DNA from oxidative stress damage [14]. In rat primary mesencephalic cultures a significantly increased ROS production and Nox subunit protein expression were observed and as early as 24 h after administration of 6-hydroxydopamine (6-OHDA), a classical toxin-inducing PD model. Furthermore, the Nox subunities gp91^phox^ and p47^phox^ were intensely expressed in microglial cells [15,16]. In line with these findings, degeneration of DA neurons induced by 1-methyl-4-phenyl-1,2,3,6-tetrahydropyridine (MPTP) was attenuated in gp91^phox-/-^ mice in comparison to the Wt mice [17].

In the present study, we explored the impact of Nox-2 in modulating inflammatory-mediated microglial responses in the 6-OHDA-induced PD model. Hereby we present critical evidence that inhibition of microglial cells with minocycline in gp91^phox-/-^ mice increases the susceptibility of these mice to develop PD through nuclear factor kappa B (NF-κB) activation and tumor necrosis factor alpha (TNF-α) release into SN.

## Materials and Methods

### Animals

Ten week-old male gp91^phox-/-^ mice (Jackson Laboratories, Maine, USA) (*n* = 60), along with wild type (Wt) mice (C57BL/6) (*n* = 70) were used throughout this study. The animals had free access to food and water and were maintained on a 12: 12 h light–dark cycle. Experiments were performed with age- and weight-matched (25-30g) animals. All procedures were approved by the Institutional Animal Care Committee of the Institute of Biomedical Sciences, University of São Paulo.

### Surgical procedures

In order to lesion the nigrostriatal system, 6-OHDA was unilaterally injected into the right striatum of both gp91^phox-/-^ and Wt mice. The animals were anaesthetized using 2-2-2 tribromoethanol (2%, Sigma-Aldrich Co., St. Louis, MO, USA) and placed into a stereotaxic frame with nose and ear bars specially adapted for mice. 6-OHDA (Sigma Chemical Co., St. Louis, MO, USA) was dissolved at a concentration of 10 µg/µl in saline (NaCl 0.9%) with 0.1% ascorbic acid [18]. The injection was performed using a Hamilton syringe (model 701) at the following coordinates: AP: −0.4 mm; ML: ±2.0 mm; DV: 3.0 mm relative to the bregma [19]. The total volume injected was 1 µl. The injection was conducted at a rate of 0.5 µl/min and the needle was left in place for additional 3 min before it was slowly removed. The left striatum received 1µl of vehicle (saline in 0.1% ascorbic acid) in the same coordinates and was used as a control. Additionally, sham-operated mice were infused with 1 µl of vehicle into both right and left striatum and served as controls in the apomorphine-induced rotation test. Clinical signs were also monitored daily after the surgery, including general body condition and dehydration. Behavioral analyses were typically conducted during the morning hours. The animals were euthanized for analysis 15 days after the surgery.

### RNA isolation, cDNA synthesis and real-time PCR

Tissue from the SN and striatum from Wt mice were directly homogenized in 1 ml TRIzol (Invitrogen, Carlsbad, CA, USA) and total RNA was isolated following the manufacturer’s suggested protocol. Following two chloroform extraction steps, RNA was precipitated with isopropanol and the pellet washed twice in 70% ethanol. After air-drying, RNA was resuspended in DEPC-treated water and the concentration of each sample obtained from *A*_260_ measurements. Residual DNA was removed using DNase I (Amersham, Piscataway, NJ, USA) by following the manufacturer’s protocol. For each 20 µl reverse transcription reaction, 2 µg total RNA was mixed with 1 µl oligodT primer (0.5 µg; Invitrogen) and incubated for 10 min at 65 °C. After cooling on ice the solution was mixed with 4 µl 5× first strand buffer, 2 µl of 0.1 M DTT, 1 µl of dATP, dTTP, dCTP and dGTP (10 mM each), and 1 µl SuperScript II reverse transcriptase (200 U; Invitrogen) and incubated for 60 min at 42 °C. Reaction was inactivated by heating at 70 °C for 15 min.

PCR reactions were performed, recorded, and analyzed using the Corbett Research system (Corbett Life Sciences, Sydney, Australia). The conditions for PCR were as follows: 50 °C for 2 min, 95 °C for 2 min, then 30 cycles of 95 °C for 15 s, 60 °C for 1 min, and 72 °C for 15 s. The specificity of the SYBR® green assay was confirmed by melting-point analysis. Expression data were calculated from the cycle threshold (Ct) value using the ΔCt method for quantification [20]. Gene expression of HPRT was used for normalization. Results were expressed as percent increases. All oligonucleotides and reagents utilized in this protocol were purchased from Invitrogen, Carlsbad, CA. Sequences used were: Nox 1 (sense 5’-CCATGAGTTTTCAATGTGGGACA -3’; antisense 5’-AACCCCCACCGCAGACTT -3’), Nox 2 (sense 5’-TCAAGACCATTGCAAGTGAACAC -3’; antisense 5’-TCAGGGCCACACAGGAAAA-3’) and Nox 4 (sense 5’-TGGGCGTCCTCGGTGGAAACT -3’; antisense 5’-CAGGCGCCCAATACGGCCAA-3’). Statistical analyses were performed using Graphpad Prism (3.02).

### Western Blots

Total, cytosolic, and plasma membrane proteins were prepared as described [21]. The membranes were incubated with the following antibodies: monoclonal anti-rabbit p67^phox^ (1:1000; Chemicon), monoclonal anti-mouse Rac-1 (1:2000, ABCAM) and monoclonal anti-β-actin (1:5000; Sigma). The probed proteins were developed by using a chemiluminescent kit (ECL, Amersham Biosciences, NJ, EUA). Films were quantified by using the NIH ImageJ analysis system.

### Immunohistochemistry

Mice were deeply anesthetized with ketamine hydrochloride (100 mg/kg of body weight, i.m.) and xylazine (16 mg/kg of body weight, i.m.) and subjected to transcardiac perfusion with a buffered saline solution, followed by a fixative solution comprised of 4% paraformaldehyde (PFA) dissolved in 0.1 M phosphate buffer (PB, pH 7.4). The brains were collected, post-fixed in PFA for 4 h, and transferred to a 30% sucrose solution in PB to ensure cryoprotection, which lasted for 48h. Brain sections (30 µm) were obtained on a sliding microtome adapted for cryosectioning. The sections were incubated free-floating for 12-16 h with anti-OX42 (CD11b/c, Biosciences, CA, USA) to detect microglial cells and anti-tyrosine hydroxylase (TH, Chemicon, Temecula, CA, USA) to detect DA neurons, both diluted 1:1000 in 0.3% of Triton X-100, containing 0.05% normal goat serum. Following 3 washes of 10 min each with PB, sections were incubated for 2 h with a biotinylated secondary antibody (donkey anti-mouse IgG, Jackson ImmunoResearch, PA, USA, 1:200), then with the avidin-biotin complex (1:100; ABC Elite kit, Vector Labs, Burlingame, CA, USA). After washing, the sections were reacted with 0.05% 3,3-diaminobenzidine and 0.01% hydrogen peroxide in PB. Intensification was conducted with 0.05% osmium tetroxide in water. The sections were mounted on gelatinized slides, dehydrated, cleared and coverslipped. Controls for immunostaining included the omission of the primary antibody, and its substitution for normal goat serum, which completely eliminated staining. The material was analyzed on a light microscope and digital images were collected. Semi-quantitative image analysis was performed using ImageJ software (National Institutes of Health/USA). OX42 immunostaining was evaluated in terms of optical density within 0.4 mm^2^ areas for SNpc. The mean optical density of striatum and SNpc labeled areas was compared with the mean density of neighboring, non-labeled areas in the same sections, to obtain a labeling index reflecting the mean signal-to noise ratio, as previously described [22]. The resulting indexes for the Wt and gp91^phox-/-^ groups were then compared and subjected to statistical analysis using Graphpad Prism 3.02 (GraphPad Software Inc., San Diego, CA, USA).

The numbers of TH-labeled cells in SNpc were determined by serial section analysis of micrographs obtained from stained brain sections using ImageJ. Measurements were taken from 5 different SNpc-containing sections through rostro-caudal axis of the structure (Bregma coordinates: -3.08, -3.16, -3.20, -3.40 and -3.52) [19] for each animal and the results were averaged and subjected to statistical analysis using the software Graphpad Prism 3.02 (GraphPad Software Inc., San Diego, CA, USA).

### 2.6 Apomorphine-induced rotation test

Apomorphine (Tocris Bioscience, Ellisville, MO, USA) was injected i.p. at a dose of 0.1 mg/kg [23]. Mice were placed in an automated rotometer (Rota-count, Columbus Instruments, Columbus, OH, USA) and allowed to adapt to their environment for 5 min before the rotations were recorded over 10 min. Results were expressed as number of rotations to the side contralateral to the lesion per minute.

### Minocycline treatment

To evaluate the impact of microglial cells inhibition in the 6-OHDA-induced PD, Wt and gp91^phox-/-^ mice received an i.p. injection of PBS or minocycline (40 mg/kg) [24], a broad-spectrum tetracycline antibiotic that exerts multiple anti-inflammatory effects, including microglial inhibition, 7 days before PD induction and for the following 14 consecutive days after the lesion. Minocycline (Sigma, St. Louis, MO) was dissolved in sterile water and sonicated to ensure complete solubilization.

### Determination of cytokines and chemokines in the brain tissue

Concentrations of IL-4, IL-1β, IL-2, IL-10, IFN-γ, TNF-α and RANTES were quantified in brain tissue samples using a mouse multiplexed bead-based immunoassay Milliplex Map Kit, MCYTOMAG-70K-PX32 (Millipore, Billerica, MA, USA) [25]. The concentration of TGF-β1 was quantified separately using a TGF-β1 Single Plex with a filter plate (non-magnetic beads) Milliplex Map Kit, TGFB-64K-01 (Millipore, Billerica, MA, USA). Briefly, SN and striatum from lesioned (6-OHDA) and anatomically matching tissue from the contralateral control hemisphere (saline) were collected from treated and non-treated Wt and gp91^phox-/-^ minocycline mice groups. The flash-frozen brain tissue was homogenized in a buffer containing 20 mmol/L Tris-HCl (pH 7.5), 150 mmol/L NaCl, 1 mmol/L PMSF, 0.05% Tween-20, and a cocktail of protease inhibitors (Roche). The protein concentration was measured in each sample. Each assay plate layout consisted of six standards in duplicate, two blank wells and up to 78 tissue samples. At the time of the assay, samples were thawed on ice and centrifuged at 20,000×g for 2 min at 4°C and the supernatant used for the analysis.

Cytokine and chemokine concentrations were determined using antibodies for each analyte covalently immobilized to a set of microspheres according to protocol developed and validated at LINCO Research, Inc [26]. The analytes on the surface of microspheres were then detected by a cocktail of biotinylated antibodies. Following binding of streptavidin–phycoerythrin conjugate, the reporter fluorescent signal was measured with a Luminex100 reader (Luminex Corp., Austin, TX 78727, USA). Data were calculated using a calibration curve obtained in each experiment using the respective recombinant proteins diluted in a lysis buffer for tissue samples. Concentration of cytokines were calculated using StatLIA® software (Brendan Scientific Corp., Calrsbad, CA 92008, USA) with a five-parameter logistic curve-fitting method, and normalized to the amount of protein in each sample. The concentration of IL-4 was below detectable levels.

### Electrophoretic mobility shift assay (EMSA) to NF-κB consensus oligonucleotide

Fifteen days after PD induction, mice were decapitated, their brains were removed and the striatum and SN samples were immediately collected. The samples were frozen in liquid nitrogen and stored at -70°C until use. Each sample was homogenized using a Dounce homogenizer in cold PBS supplemented with 0.5 mM DTT, 0.5mM PMSF, 2 lg/ml leupeptin, 2 lg/ml antipain, and 3 mM sodium ortovanadate and centrifuged at 48C for 30 sec at 12,000g. Pellets were resuspended in lysis buffer (10 mM HEPES pH 7.9, 1.5 mM MgCl2, 10 mM KCl, 0.1 mM EDTA, 0.5 mM PMSF, 2 µg/mL leupeptin, 2 µg/mL antipain, 3 mM sodium ortovanadate, 30 mM sodium fluoride, 20 mM sodium pyrophosphate) and incubated on ice for 10 min. After the addition of NP-40 (0.5%), samples were mixed and centrifuged for 30 s at 13,000 g. Nuclei were resuspended in extraction buffer (20 mM HEPES, pH 7.9, 25% glycerol, 1.5 mM MgCl2, 300 mM NaCl, 0.25 mM EDTA, 0.5 mM PMSF, 2 µg/mL leupeptin, 2 µg/mL antipain), incubated for 20 min on ice and centrifuged for 20 min at 13,000 g at 4°C. The remaining supernatants containing nuclear proteins were stored at -80°C. Protein concentration was determined using the BioRad protein reagent. EMSA to NF-κB was performed using a gel shift assay kit from Promega [27-29]. NF-κB double-stranded consensus oligonucleotide (5'-AGTTGAGGGGACTTTCCCAGGC- 3') was end-labeled with γ-^32^P-ATP using T4 polynucleotide kinase. Unincorporated nucleotides were removed by running the reaction mixture through a Sephadex G-25 spin column (Amersham-Pharmacia, Uppsala, Sweden). Purified ^32^P-labeled probe (30,000 cpm) was incubated in 20 µL with 5 µg nuclear extracts in a binding reaction mixture containing 50 mM NaCl, 0.2 mM EDTA, 0.5 mM DTT, 4% glycerol, 10 mM Tris-HCl (pH 7.5) and 0.05 µg poly (dI-dC) for 30 min at room temperature. DNA-protein complexes were separated by electrophoresis through a 6% non-denaturing acrylamide:bis-acrylamide gel in 0.5×Tris-borate/EDTA (TBE) for 2h at 150V. Gels were vacuum-dried and analyzed by autoradiography. Autoradiograph quantification was performed with ImageJ (National Institutes of Health/USA). Previous studies of our laboratory ran competition experiments in brain control samples by using NF-κB and TFIID (5´-GCAGAGCATATAAGGTGAGGTAGGA-3`) unlabeled double-stranded consensus oligonucleotide in five- to 20- fold molar excess over the amount of ^32^P- NF-κB probe in order to detect specific (NF-κB) and non-specific DNA-protein interactions (NS), respectively. To the specificity of the assay, unlabeled oligonucleotides were added to the reaction mixture 20 min before the radioactive probe [30,31].

### Data analysis

Data were expressed as means ± standard error of the mean (SEM) and were analyzed using two-way analysis of variance (ANOVA), followed by pairwise comparisons (Tukey’s HSD test). For individual comparisons, statistical analysis was performed using unpaired Student’s *t*-test. In all cases, *p* ≤ 0.05 was considered to be statistically significant.

## Results

### NADPH oxidase activation in the 6-OHDA-induced PD mice model

**Figure 1:**
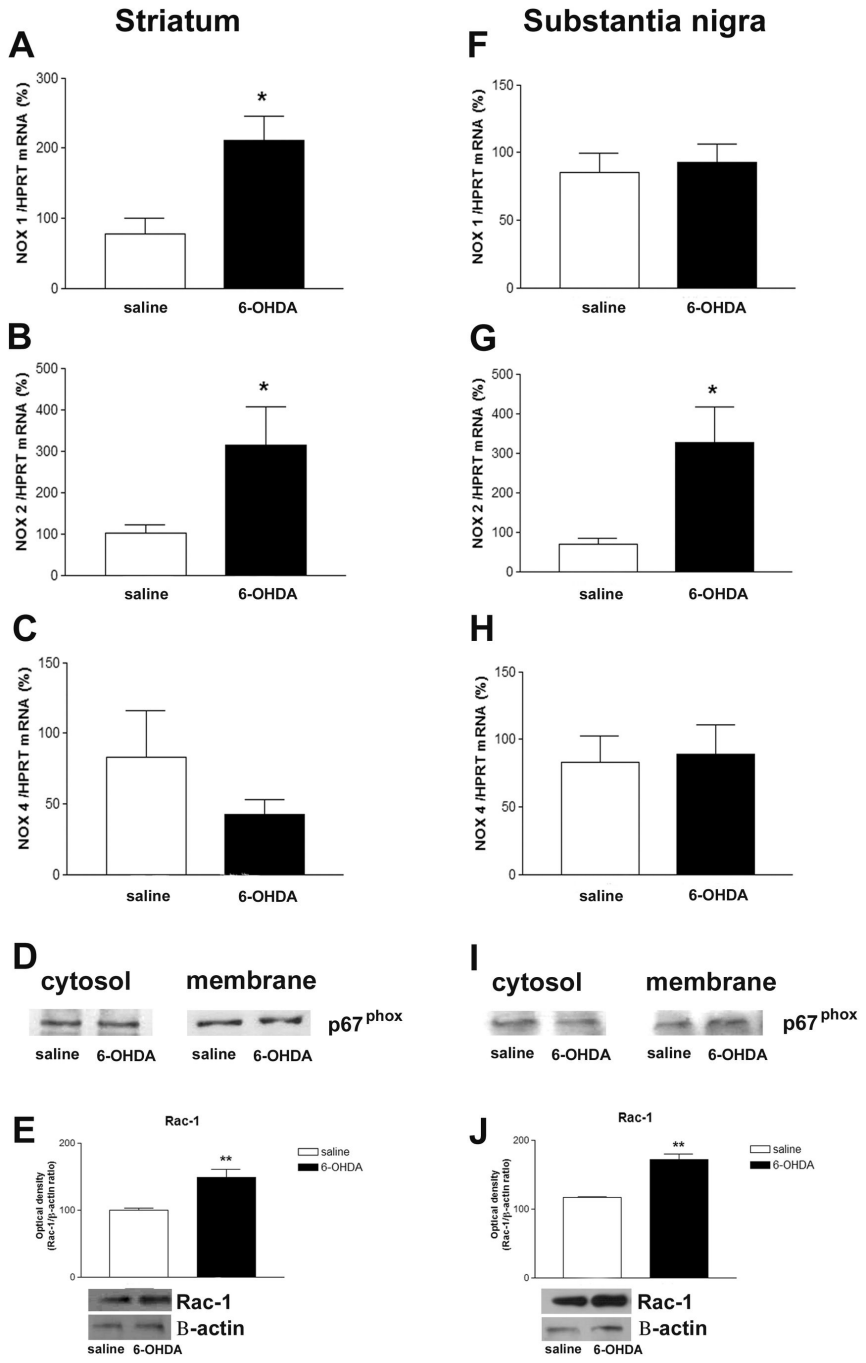
Six-OHDA leads to Nox activation. Effect of 6-OHDA on mRNAs of Nox1 (A), Nox2 (B) and Nox4 (C) isoforms in the striatum. HPRT was used as an internal control; n=6. The statistical significance is expressed as: * P < 0.05. In D and I, representative Western blots illustrating p67^phox^ protein content in cytosolic and membrane fractions obtained from striatum and SN respectively, after 6-OHDA-induced PD. Six-OHDA did not induce significant p67^phox^ translocation from the cytosol to the plasma membrane in none of the analyzed samples; n=4 tested samples. In E and J, effect of 6-OHDA on striatal and SN Rac-1 total protein levels. The graphs represent the mean ratio of Rac-1 densitometric data in relation to β-actin of 5 tested samples. Significance compared to Wt saline at **p <0.01. In F, effect of 6-OHDA on mRNAs of Nox1 (F), Nox2 (G) and Nox4 (H) isoforms in the SN. HPRT was used as an internal control; n=6. The statistical significance is expressed as: * P < 0.05.

In order to test whether Nox isoform gene expression can be induced by 6-OHDA, the mRNAs encoding each Nox isoforms were evaluated by RT-PCR in striatum and SN after the lesion. Nox1, Nox2 and Nox4 isoforms were detected in both structures analyzed. Nox1 and Nox2 gene expression were found to be increased in striatum (Figure 1 A-C), whereas enhanced Nox2 gene expression was observed into SN (Figure 1F-H). Classically, activation of Nox2 requires the translocation of its cytosolic subunits to plasma membrane. In 6-OHDA injected Wt mice, Western blotting assays revealed no alterations of p67^phox^ membrane and cytosolic protein content in either striatum or SN (Figure 1D and 1I). However, our Western blotting assays clearly showed increased Rac-1 (a small GTP binding protein required for the assembly of the Nox complex) total protein content in both structures analyzed (Figure 1E and 1J).

### The NADPH oxidase complex is involved in 6-OHDA-mediated dopaminergic degeneration

**Figure 2:**
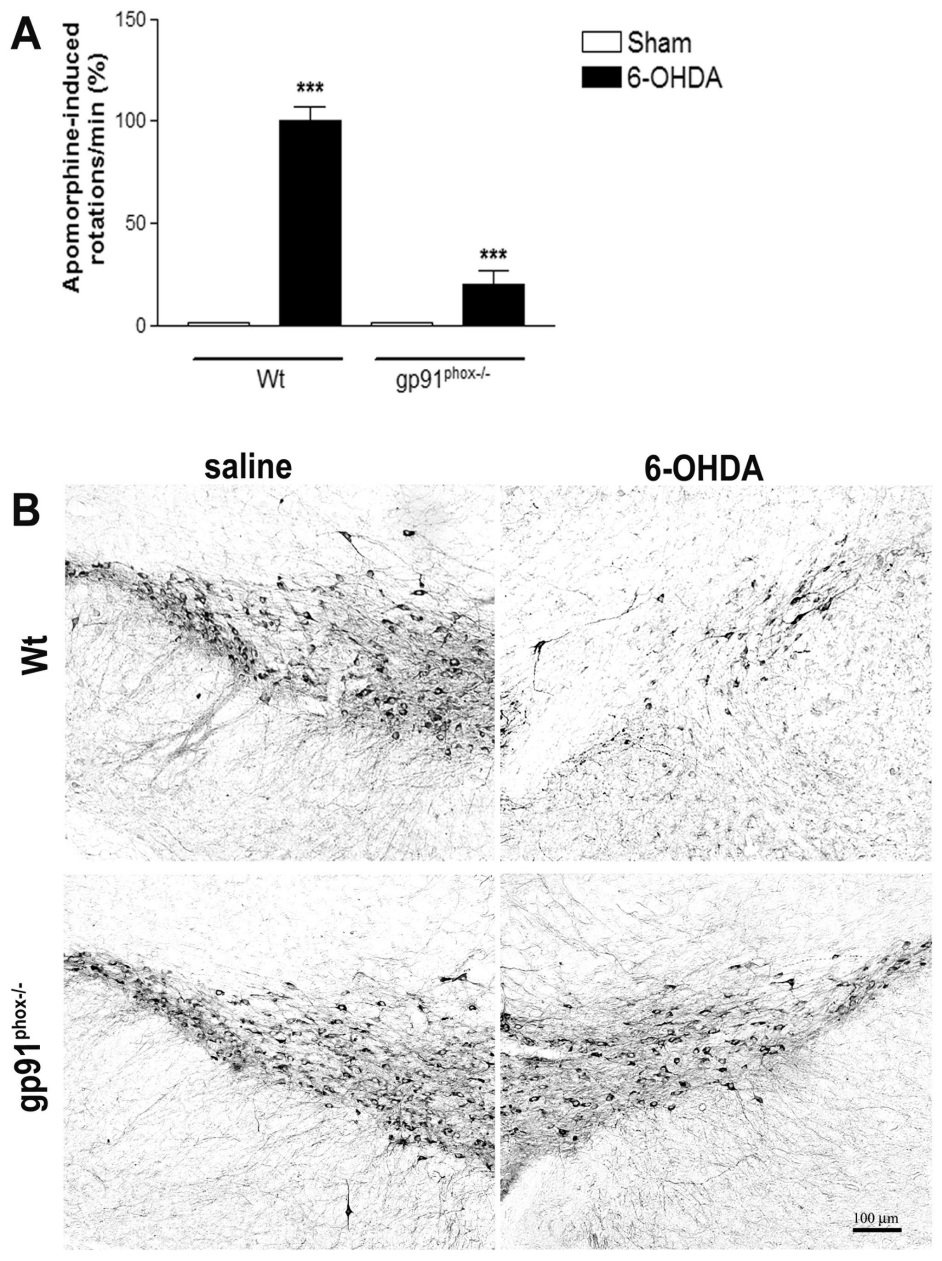
NADPH oxidase is involved in the 6-OHDA-mediated degeneration of DA neurons in SN. A, apomorphine-induced contralateral rotations evaluated at 14 days post-PD induction in Sham, Wt 6-OHDA-lesioned mice and gp91^phox-/-^ 6-OHDA lesioned mice groups; n=12. Values are expressed as percent of induced rotations/min in relation to the Sham group. Representative digital images demonstrating TH-immunoreactive cell bodies in the SN of Wt saline, Wt 6-OHDA and gp91^phox-/-^ 6-OHDA-lesioned mice groups (B). Images are representative of at least six independent experiments. The statistical significance is expressed as: *** P<0.001 *versus* Wt Sham group and *** p<0.001 gp91^phox-/-^ 6-OHDA *versus* Wt 6-OHDA.

Apomorphine-induced rotation tests were performed at day 14th post-lesion. Administration of apomorphine was found to stimulate contralateral rotational behavior in Wt mice (p<0.001), when compared to the Sham group. Gp91^phox-/-^ 6-OHDA-lesioned mice exhibited a significant reduction in the apomorphine-induced rotational response (80% *vs* Wt 6-OHDA-lesioned mice, p<0.001) (Figure 2A). TH-immunolabeling indicated that unilateral striatal 6-OHDA injections in wt mice reduced the number of DA neurons in the SN, in comparison to the saline group. In contrast, in gp91^phox-/-^ 6-OHDA-lesioned mice the DA degeneration was negligible (Figure 2B), further supporting our hypothesis that Nox2 play a role in the degeneration of DA neurons in PD.

### Minocycline treatment alters the rotational behavior and TH immunostaining in gp91^phox-/-^-lesioned mice

**Figure 3:**
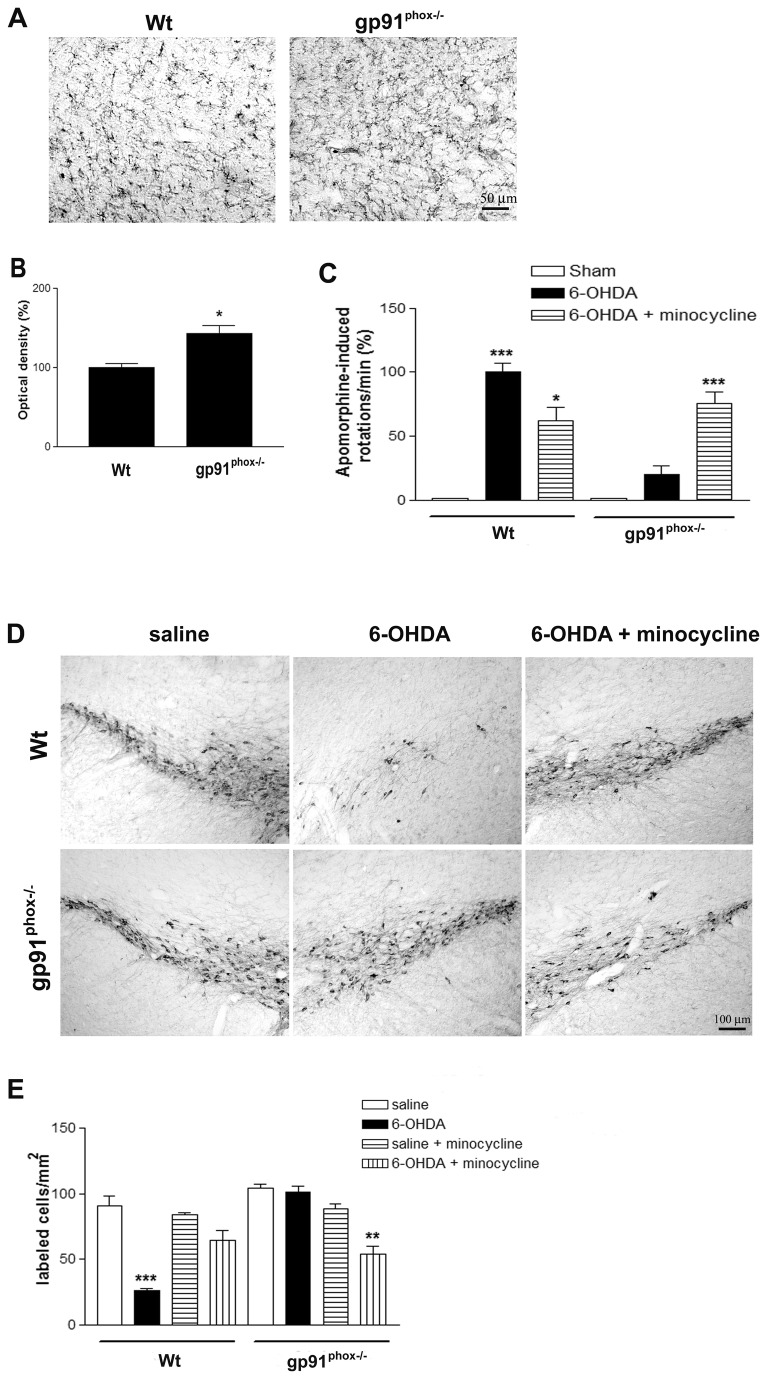
Minocycline treatment alters the rotational behavior and the SN TH-immunostaining of 6-OHDA-lesioned mice. Representative digital images of OX42 immunoreactivity in the SN of Wt and gp91^phox-/-^ control mice (A). The graph represents OX42 optical density analysis; n=6 (B). The statistical significance is expressed as: * P<0.05. C, apomorphine-induced rotational behavior evaluated after the minocycline treatment in Sham, Wt 6-OHDA-lesioned mice, Wt 6-OHDA-lesioned mice treated with minocycline, gp91^phox-/-^ 6-OHDA lesioned mice and gp91^phox-/-^ 6-OHDA lesioned mice treated with minocycline groups; n=8-10. Values are expressed as the percent of induced rotations/min in relation to the Sham group. The statistical significance is expressed as: *** P<0.001 *versus* Sham group, * P<0.05 *versus* Wt 6-OHDA-lesioned group and *** P<0.001 *versus* gp91^phox-/-^ 6-OHDA lesioned group. D, representative digital images of TH immunoreactivity in Wt saline, Wt 6-OHDA-lesioned mice, Wt 6-OHDA-lesioned mice minocycline, gp91^phox-/-^ saline, gp91^phox-/-^ 6-OHDA-lesioned mice and gp91^phox-/-^ 6-OHDA lesioned mice minocycline groups. E the graph depicting cell counts of TH immunostaining in the SN. The statistical significance is expressed as: *** P<0.001 *versus* Wt saline group and ** P<0.01 *versus* gp91^phox-/-^ saline group (n=6-8).

By using standard immunostaining procedures, we compared OX42 immunoreactivity in the SN of Wt and gp91^phox-/-^ mice in order to investigate whether the deletion of gp91^phox^ subunit would affect microglial cells in normal conditions. The intensity of OX42 immunoreactivity and number of OX42- positive cells were higher in the gp91^phox-/-^ mice (Figure 3A and B), suggesting that deletion of gp91^phox^ subunit elevates the population of microglial cells within the SN. This observation led us to treat Wt and gp91^phox-/-^ mice with the microglia inhibitor minocycline to determine whether microglial cells play detrimental or beneficial roles in gp91^phox-/-^ mice after PD induction. After the treatment, apomorphine-induced rotation tests were performed to characterize the impact of lesion induced by 6-OHDA and the minocycline effects. As expected, the treatment with minocycline was able to significantly decrease the number of contralateral rotations per minute in Wt-6-OHDA-lesioned mice (40%, p<0.05). However, the quantification of rotational behavior in gp91^phox-/-^ lesioned mice showed increased susceptibility of gp91^phox-/-^ lesioned mice treated with minocycline to develop apomorphine-induced rotational behavior (p<0.01 - gp91^phox-/-^ 6-OHDA lesioned vs gp91^phox-/-^ 6-OHDA lesioned treated with minocycline). Wt 6-OHDA lesioned mice and gp91^phox-/-^ lesioned mice treated with minocycline showed similar rotation scores over repeated tests, suggesting that microglial cells and Nox2 are involved in the signaling pathways underlying the inflammatory response (Figure 3C). Corroborating with these data, we also observed that minocycline treatment reduced the degeneration of TH-positive neurons induced by 6-OHDA in Wt mice. Nevertheless, the same treatment was able to significantly decrease the number of TH-positive neurons in gp91^phox-/-^ 6-OHDA-lesioned mice (Figure 3D and E), suggesting an enhancement of neurodegeneration.

### Minocycline treatment increases TNF-α release in the substantia nigra of gp91^phox-/-^-lesioned mice

The next series of experiments investigated the mechanism by which the minocycline treatment increases the susceptibility of gp91^phox-/-^ 6-OHDA lesioned mice to develop PD. We analyzed the concentration of multiple cytokines and one chemokine in SN and striatum samples from control Wt and gp91^phox-/-^ mice and in 6-OHDA-lesioned mice treated with minocycline or PBS. All the molecules measured have been previously implicated in the pathogenesis of PD and included proinflammatory cytokines (IL-1β, TNF-α, IFN-γ, IL-2), anti-inflammatory cytokines (IL-10 and TGF-β1) and the chemokine RANTES.

**Figure 4:**
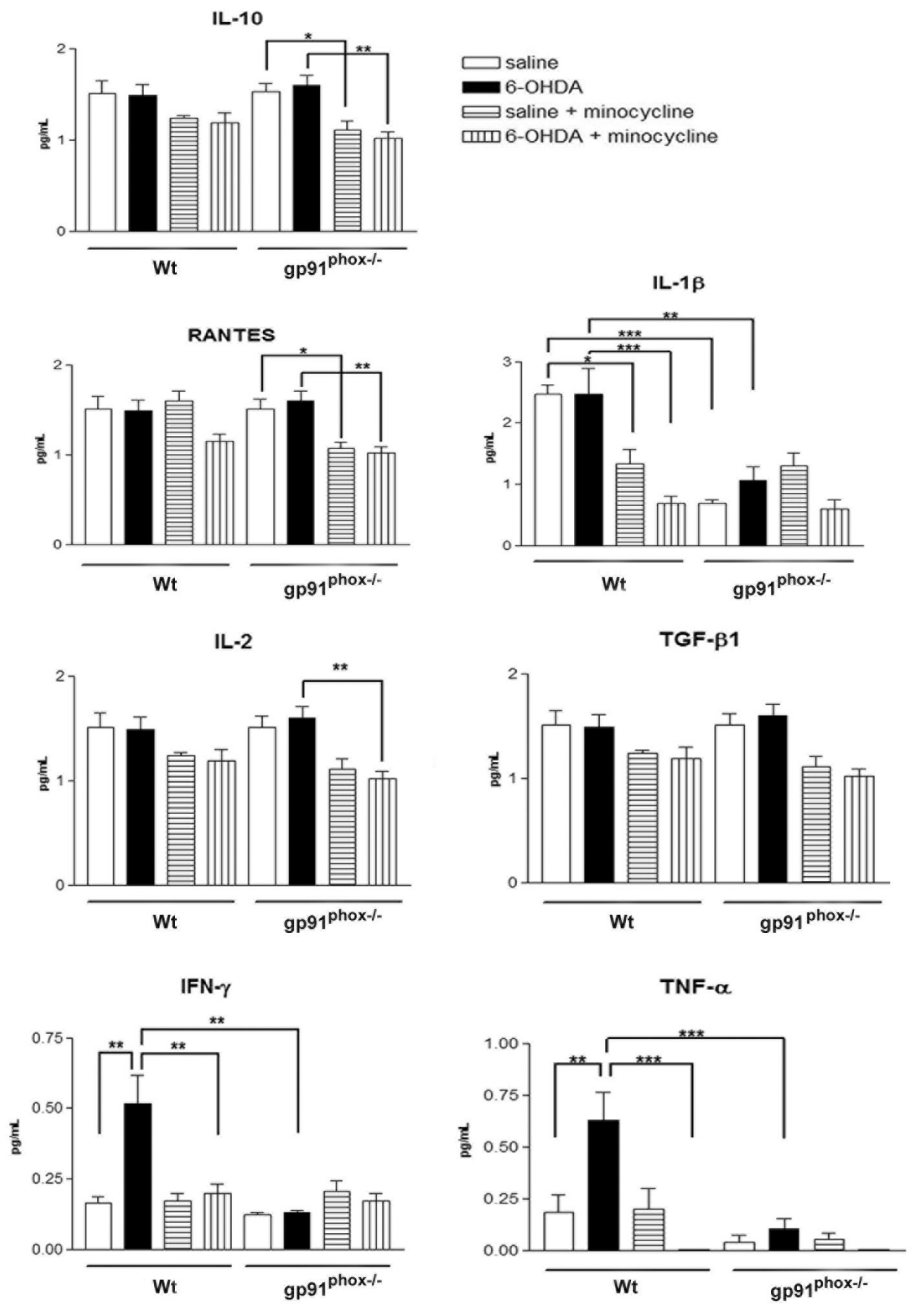
Effect of minocycline treatment on striatal cytokine concentration in Wt and gp91^phox-/-^ 6-OHDA-lesioned mice. Striatal concentration of pro-inflammatory (IL-1β, TNF-α, IFN-γ, IL-2) and anti-inflammatory cytokines (IL-10 and TGF-β1) and the chemokine RANTES following 6-OHDA-induced PD in Wt and gp91^phox-/-^ treated with minocycline or PBS. All concentrations were expressed in pg/ml. The statistical significance is expressed as: * P<0.05; ** P<0.01 and *** P<0.001; n=6 for each experimental group tested.

In striatum, 6-OHDA was able to significantly increase the production of IFN-γ and TNF-α, which was not observed in gp91^phox-/-^ mice. Striatal concentration of IL-10, RANTES, IL-1β, IL-2 and TGF-β1 remained the same after the lesion in both Wt and gp91^phox-/-^ mice. The basal concentration of IL-1β was found significantly diminished in gp91^phox-/-^ mice, when compared to Wt mice. Minocycline treatment significantly decreased the production of IFN-γ and TNF-α stimulated by 6-OHDA. A trend towards a decreased production of all the cytokines analyzed was observed after the minocycline treatment, although, the treatment was only able to significantly decrease the basal production of IL-10, RANTES and IL-2 in gp91^phox-/-^ mice and of IL-1β in Wt mice (Figure 4).

**Figure 5:**
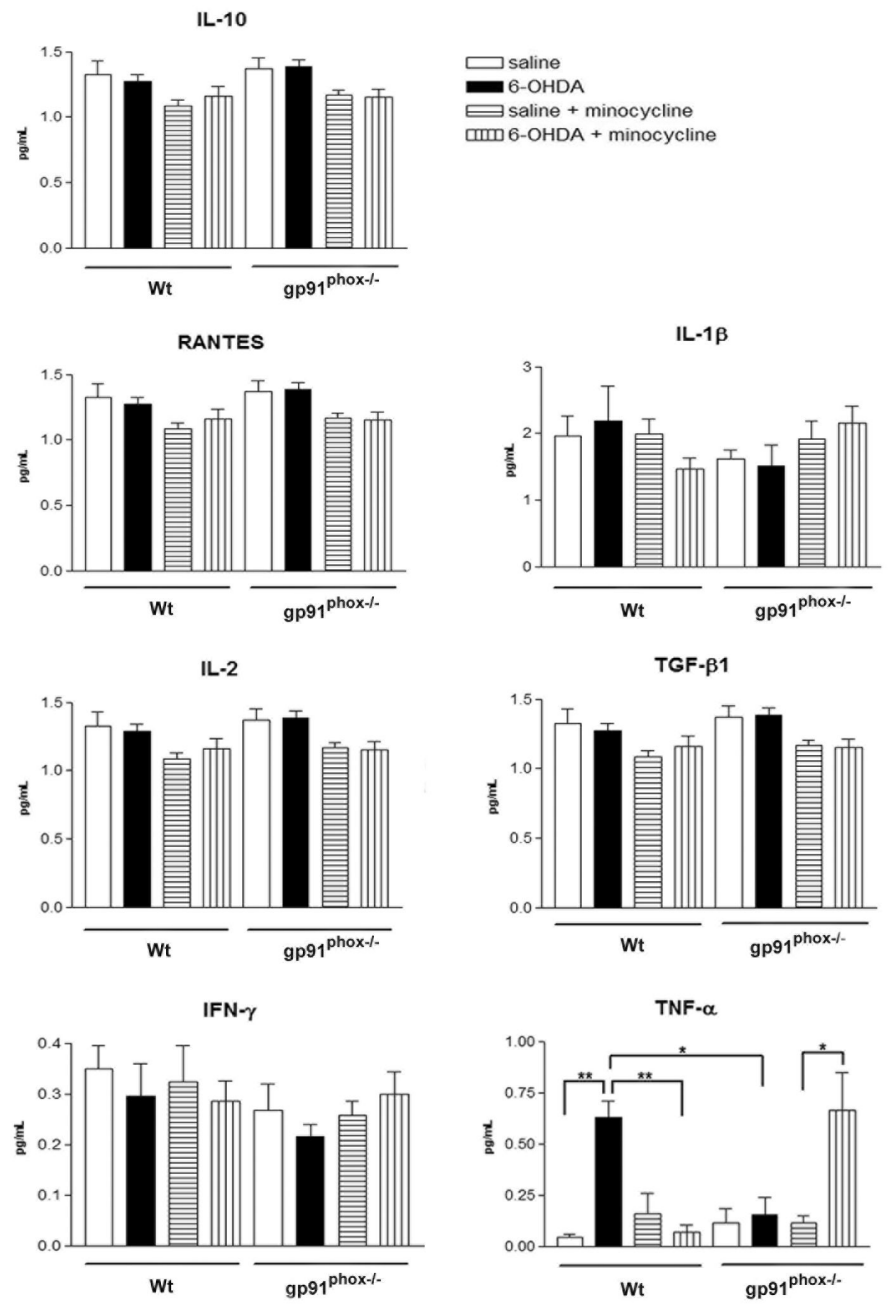
Effect of minocycline treatment on substantia nigra cytokine concentration in Wt and gp91^phox-/-^ 6-OHDA-lesioned mice. Concentration of pro-inflammatory (IL-1β, TNF-α, IFN-γ, IL-2) and anti-inflammatory cytokines (IL-10 and TGF-β1) and the chemokine RANTES in the substantia nigra following 6-OHDA-induced PD in Wt and gp91^phox-/-^ treated with minocycline or PBS. All concentrations were expressed in pg/ml. The statistical significance is expressed as: * P<0.05; ** P<0.01 and *** P<0.001; n=6 for each experimental group tested.

In the SN, 6-OHDA did not stimulate the production of IL-10, RANTES, IL-1β, IL-2, TGF-β1 and IFN-γ. Unlike what was found in striatum samples, minocycline did not change the basal production of any of the molecules tested. The production of TNF-α was found to be significantly increased after 6-OHDA injection in Wt but not in gp91^phox-/-^ mice. However, the treatment with minocycline increased TNF-α production in gp91^phox-/-^ 6-OHDA injected mice, further supporting our hypothesis that Nox-2 is modulating inflammatory-mediated microglial responses (Figure 5).

### Minocycline treatment potentiates NF-κB activation in gp91^phox-/-^ lesioned mice

**Figure 6:**
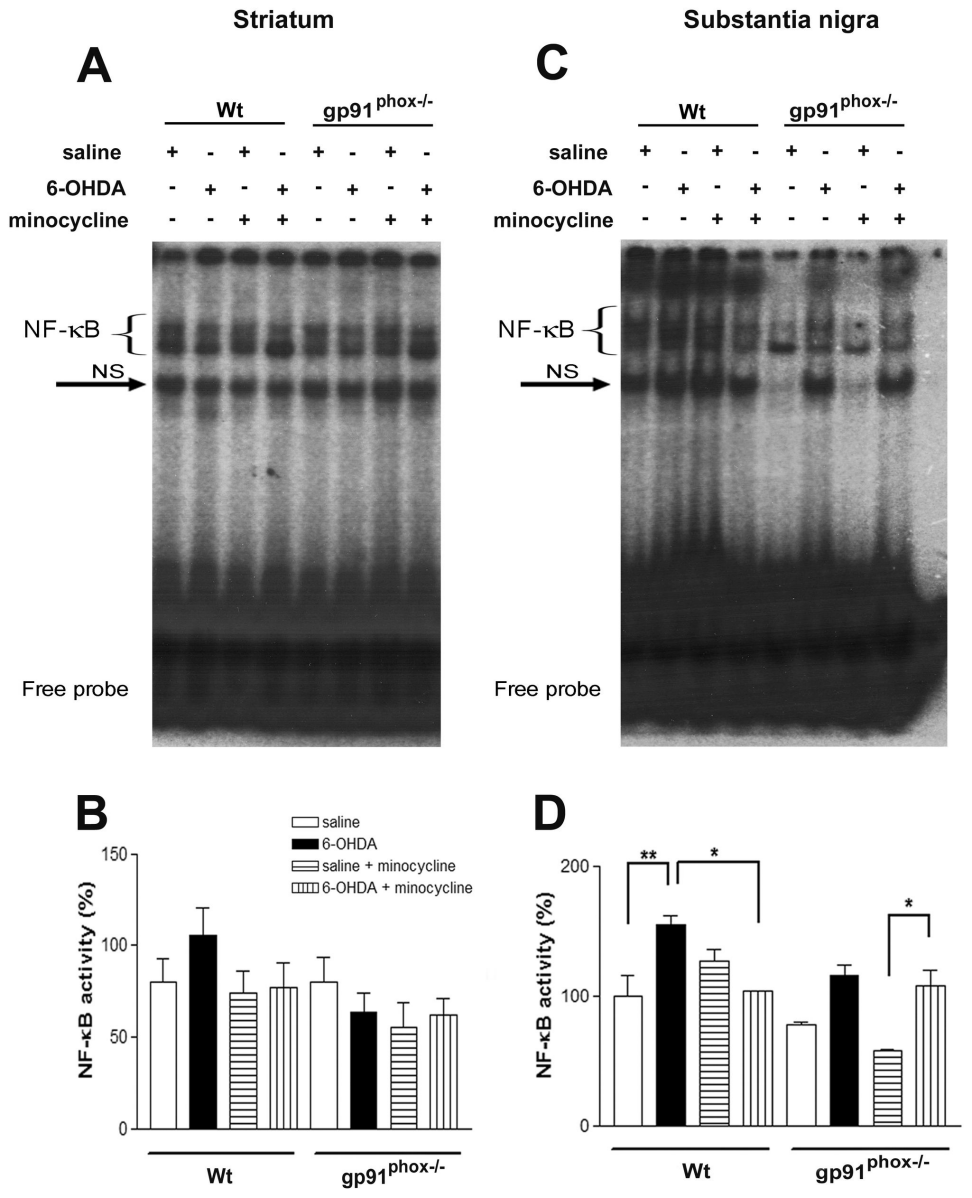
Minocycline treatment potentiates NF-κB activation in gp91^phox-/-^ 6-OHDA lesioned mice. In A, representative panel of NF-κB activation in striatal nuclear extracts. In B, densitometric analysis of NF-κB bands obtained from nuclear extracts of striatum. In C, representative panel of NF-κB activation in SN nuclear extracts. In D, densitometric analysis of the NF-κB bands obtained from nuclear extracts of SN. The statistical significance is expressed as: * P<0.05; ** P<0.01 and *** P<0.001; n=4. Competition studies were performed using brain extract (15 µg) in the absence or presence of unlabeled specific (NF-κB consensus sequence, 5-, 10- and 20- fold molar excess) or non-specific oligonucleotide (TFIID consensus sequence, 20- fold molar excess), as indicated. The position of specific NF-κB/DNA binding complexes is indicated (NF-κB). NS represents no specific binding (NS). The localization of the probe is also indicated. Results are representative of three experiments.

Since NF-κB activation induces the production and release of TNF-α, we investigate whether inhibition of microglial cells in gp91^phox-/-^ lesioned mice could modulate NF-κB activation. As evaluated by EMSA, 6-OHDA did not change basal NF-κB activity in the striatum of Wt mice (Figure 6A and B). However, 6-OHDA significantly increased NF-κB/DNA binding activity in SN nuclear extracts, which was clearly attenuated by the minocycline treatment. In gp91^phox-/-^ lesioned mice, 6-OHDA did not change basal NF-κB binding activity in either brain region, although a trend towards an increase in SN samples has been observed. However, minocycline treatment significantly potentiates 6-OHDA-induced NF-κB activation in the SN of gp91^phox-/-^ lesioned mice (Figure 6C and D), suggesting that NF-κB activation may be related to the increased release of TNF-α.

## Discussion

The present study investigated the impact of Nox-2 in the modulation of inflammatory-mediated microglial responses in the 6-OHDA- induced PD. Our major findings demonstrate that (1) The Nox2 isoform is involved in 6-OHDA-mediated DA degeneration in SN; (2) inhibition of microglial cells by the treatment with minocycline increases the susceptibility of gp91^phox-/-^ 6-OHDA lesioned mice to develop PD, as evaluated by apomorphine-induced rotational behavior and TH immunolabeling; (3) minocycline treatment leads to NF-κB activation and TNF-α release into the SNpc of gp91^phox-/-^ 6-OHDA lesioned mice.

Minocycline, a tetracycline derivative, is a versatile drug with a broad spectrum of action including multiple anti-inflammatory effects, besides its anti-microbial properties. In the central nervous system, it has been demonstrated that minocycline inhibits apoptosis, proteolysis and the activation and proliferation of microglial cells, triggered by several different stimuli [32]. However, the molecular mechanisms underlying its anti-inflammatory actions and its influence on cytokine release are not fully understood [33]. Minocycline has been reported to reduce dopaminergic degeneration, due to an attenuation of microglial cell activation following unilateral 6-OHDA injection into striatum of mice [34]. Here we investigated minocycline ability (through microglial cell inhibition) to change the susceptibility of gp91^phox-/-^ 6-OHDA lesioned mice to develop PD.

Overactivation of Noxes leads to excessive ROS production, which disrupts redox signaling and results in oxidative stress [35]. Independently of the ROS source, oxidative stress is increasingly recognized as one of the most relevant contributors to neurodegeneration [36,37].

Although the exact etiology of PD still remains largely unknown, some studies have implicated increased ROS production through Noxes in the pathophysiology of this disease [38-40]. Current research also suggests that DA neurons are inherently more vulnerable to oxidative stress, when compared to other cell types [41]. Consistent with previous reports, we observed that 6-OHDA induces increased gene expression of Nox1 and Nox2 isoforms [14,15], suggesting that Nox activation is a relevant component of the 6-OHDA-induced DA degeneration. Corroborating with this view, a strong argument for a role of Nox2 activation in neuronal death is the fact that gp91^phox-/-^ exhibited significantly reduced apomorphine-induced rotational behavior and also a negligible degeneration of DA neurons after 6-OHDA-injections.

Over the last decades the question of whether microglial cells play detrimental or beneficial roles in neurodegenerative conditions has been widely debated [42]. Similar to peripheral macrophages, microglial cells are functionally polarized into different activation phenotypes during neuroinflammation. It has been recently demonstrated that Nox plays a critical role in the modulation of the microglial phenotype. In fact, hippocampal levels of IL-4, an anti-inflammatory cytokine, and the expression of IL-4 receptor α mRNA were significantly increased 24 h after intracerebroventricular injection of LPS in p47^phox-/-^ mice when compared to wt mice, indicating that deletion of p47^phox^ subunit alters the IL-4-dependent signaling pathway and attenuates the inflammatory response [14].

Despite the fact that the deletion and the pharmacological inhibition of the Nox complex promotes an anti-inflammatory microglial activation after LPS injection, we present here strong evidence that Nox2 plays an important role in modulating the inflammatory response induced by 6-OHDA through a TNF-α/NF-κB mediated signaling pathway, a likely mechanism whereby gp91^phox-/-^ 6-OHDA lesioned mice may be more susceptible to develop PD after microglial cell inhibition. The cooperative relationship between TNF-α production, a classic inflammatory cytokine, and NF-κB activation has been previously demonstrated as an important molecular event occurring in the 6-OHDA-induced neurodegeneration [43,44].

TNF-α release has been identified as a critical mechanism involved in DA neuroinflammation and in neuron damage. Continuous production of this cytokine induced by an adenoviral vector leads to chronic microglia and macrophage activation in the SN, progressive neurodegeneration, and delayed motor symptoms [45]. Among all the cytokines evaluated in the present study, we observed that microglial cell inhibition increased TNF-α production into the SN of gp91^phox-/-^-6-OHDA lesioned mice. It is noteworthy that astrocytes are able to release TNF-α [46], which could be one possible explanation for the increased TNF-α concentration in the SN of gp91^phox-/-^-6-OHDA lesioned mice after microglial cell inhibition observed in the present study. Increased LPS-induced TNF-α release from astrocyte cultures has been demonstrated [47], and increased astrocytic immunoreactivity for TNF-α was observed in the SN of parkinsonian patients [48]. In addition, in parkinsonian monkeys TNF-α immunoreactivity was observed very close to GFAP immunoreactivity in activated astrocytes, which suggests that TNF-α synthesis and/or release may be intimately linked with the astrocyte cytoskeleton [49].

We also observed in gp91^phox-/-^ 6-OHDA lesioned mice that minocycline treatment potentiated the activation of NF-κB, which can induce TNF-α production [50], suggesting the participation of that transcription factor in the process. However, considering that TNF-α release may also trigger a signaling cascade that can converge on the activation of the transcription factor NF-κB [51], whether NF-κB activation precedes TNF-α production is an issue that remains to be determined.

Since inhibition of microglial cells by minocycline treatment increased the susceptibility of gp91^phox-/-^ 6-OHDA lesioned mice to develop PD and exacerbated the pro-inflammatory response induced by the neurotoxin, our results further strengthen the hypothesis that Nox2 adds an essential level of regulation to signaling pathways underlying the inflammatory response after PD induction. The Nox complex may play a role not only in the signaling events leading to microglia activation, but may also act as an important modulator of signal transduction in other cell types. Our results are also likely to provide further insights in the direction of a better understanding of the mechanisms of Nox-dependent oxidative stress involvement in pathophysiological conditions.

## Conclusions

We demonstrated here that inhibition of microglial cells in gp91^phox-/-^ 6-OHDA lesioned mice triggers DA degeneration and exacerbated the pro-inflammatory response induced by the neurotoxin through a TNF-α/NF-κB mediated signaling pathway. Our results suggest that Nox2 adds an essential level of regulation to signaling pathways underlying the inflammatory response after PD induction, and indicated that both Nox2 and microglial cells could represent therapeutics targets in PD.
